# Outcomes of Living Donor Liver Transplantation in Patients With a History of Ruptured Hepatocellular Carcinoma

**DOI:** 10.3389/fsurg.2021.722098

**Published:** 2021-10-18

**Authors:** Hwi Yeol Lee, Suk Kyun Hong, Su young Hong, Sanggyun Suh, Eui Soo Han, Jeong-Moo Lee, YoungRok Choi, Nam-Joon Yi, Kwang-Woong Lee, Kyung-Suk Suh

**Affiliations:** Department of Surgery, Seoul National University College of Medicine, Seoul, South Korea

**Keywords:** ruptured hepatocellular carcinoma, tumor recurrence, living donor liver transplantation (LDLT), liver transplant, survival

## Abstract

**Background:** Liver transplantation (LT) is considered a contraindication in patients with a history of hepatocellular carcinoma (HCC) rupture because ruptured HCCs are classified as T4 in the current American Joint Committee on Cancer TNM system. This study aimed to assess living donor liver transplantation (LDLT) in these patients and elucidate the factors that may have affected their outcomes.

**Methods:** Data of patients with a history of ruptured HCC who underwent LDLT between January 1999 and December 2019 were retrospectively reviewed.

**Results:** Among 789 patients who underwent LDLT for HCC, five (0.64%) had a history of HCC rupture. Three patients (60%) were treated with transarterial chemoembolization (TACE) or transarterial embolization (TAE) for hemostasis, and two patients (40%) achieved spontaneous hemostasis. One of two patients who achieved spontaneous hemostasis underwent surgical resection and LT at 1 week and 6 years after the rupture, respectively. The other patient underwent LT 2 days after the rupture. Four patients (80%) survived for >5 years, while two patients (40%) experienced recurrence and succumbed during the median follow-up duration of 85.3 months (range, 12.4–182.7). The recurrence first developed at 4.3 and 17.0 months after LT; these patients were managed well using surgical resection for peritoneal seeding and TACE for intrahepatic HCC.

**Conclusion:** LDLT can be considered a treatment method even in patients with a history of HCC rupture after full evaluation of tumor biology and risk of recurrence.

## Introduction

Spontaneous rupture of hepatocellular carcinoma (HCC) is a life-threatening complication that can occasionally occur due to hypervascularity. The incidence rate of spontaneous rupture of HCC has recently decreased from 3–13 to 2.3–5.9% (1, 2) due to advances in surveillance protocols and imaging modalities (3, 4). HCC rupture can cause massive bleeding, leading to a high mortality rate (25–75%) (4–6) and may increase the risk of peritoneal dissemination (7). Therefore, the current American Joint Committee on Cancer TNM system classifies all ruptured HCC cases as T4 (8).

Cases of ruptured HCC may be treated in palliative settings using transarterial chemoembolization (TACE) rather than curative treatment. However, resection and TACE are widely performed in patients with a history of spontaneous rupture of HCC, as previous studies have shown favorable long-term outcomes (9). Most patients with ruptured HCC either have poor liver function or are not candidates for curative surgical resection upon detection. However, many studies have demonstrated that rupture is not a prognostic factor for surgical outcomes (5, 10, 11). Moreover, some studies have reported that spontaneous rupture of HCC did not increase the risk of peritoneal dissemination (10, 11).

For curative treatment in cases of ruptured HCC, most studies preferred partial hepatectomy after primary bleeding control of tumor rupture with TACE. However, there have been few reports on liver transplantation (LT) as a curative treatment for patients with a history of HCC rupture. There are more living donor liver transplantations (LDLTs) than deceased donor liver transplantations (DDLTs) due to an organ shortage in Korea. Therefore, our center selects patients to undergo LDLT beyond the Milan criteria based on significant preoperative biological factors and patient requests (12). In a few patients with a history of HCC rupture who made a strong request to undergo LDLT, LT was performed, risking the possibility of recurrence.

There are only a few case reports on LT in patients with HCC rupture (13–15) and without evaluation of any biological markers. We experienced favorable outcomes in some patients with selective tumor biology. Therefore, we aimed to retrospectively analyze the natural course and outcomes in patients undergoing LDLT with a history of spontaneous HCC rupture, ultimately evaluating the efficacy of LDLT as an alternative curative treatment.

## Materials and Methods

### Patients and Methods

Patients with a history of spontaneous rupture of HCC were retrospectively reviewed from the prospective data of patients diagnosed with HCC treated with LDLT between January 1999 and December 2019 at Seoul National University Hospital. Dynamic contrast-enhanced abdominal computed tomography (CT) was used for the diagnosis of spontaneous HCC rupture. The institutional review board of Seoul National University Hospital approved this study (IRB no. 2003-114-1110), and the requirement for informed consent was waived due to the retrospective nature of this study.

Clinical data, including sex, age, underlying liver disease, tumor size and number based on image, Child-Turcotte-Pugh class and score, model for end-stage liver disease (MELD) score before transplantation, presence of portal vein tumor thrombus (PVTT) on imaging, positron emission tomography (PET) results, the interval from rupture to LT, and treatment administered between rupture of HCC and LT were evaluated preoperatively. Intraoperative data of estimated blood loss, operation duration, pathologic data of size and number of tumors, percentage of necrosis, differentiation grade, and presence of microvascular invasion were analyzed. We also investigated follow-up duration and outcome, recurrence-free survival, overall survival, and treatment management after recurrence. Levels of serum alpha-fetoprotein (AFP), protein induced by vitamin K absence or antagonist-II (PIVKA-II), and immunosuppressants were reviewed during the complete treatment period.

Tumor size was defined as the largest diameter of the major tumor, regardless of the viability in both imaging and pathological analysis. Follow-up duration was defined as the interval from the date of LT to the last outpatient clinic visit. Recurrence-free survival was defined as the duration between LT and the day when recurrence was first diagnosed by imaging. Overall survival was defined as the interval from LT to the date of death or last follow-up outpatient clinic visit.

After discharge, all the patients underwent follow-up examinations every week during the first month, twice a month for 2 months, monthly during the first year, and every 3 or 4 months thereafter. Immunosuppression was initially based on a triple regimen comprising calcineurin inhibitors (tacrolimus or cyclosporine A), mycophenolate mofetil (MMF), and prednisolone. Inhibitors of the mammalian target of rapamycin (mTOR), sirolimus, or everolimus, were administered in some cases, tapering of calcineurin inhibitors. Abdominal CT or magnetic resonance imaging (MRI) was performed every 3–4 months for recurrence surveillance.

### Statistical Analysis

Results were expressed as median (range) for continuous data and as numbers with percentages for categorical data. Statistical analysis was performed using the SPSS software (version 25; SPSS Inc., Chicago, IL, USA).

## Results

**Figure 1:**
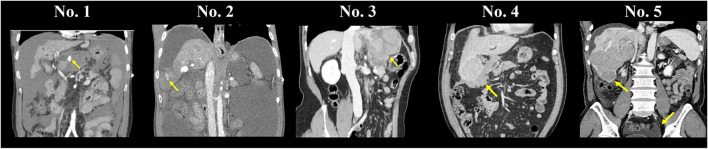
Computed tomography scan of a ruptured hepatocellular carcinoma.

A total of 786 patients diagnosed with HCC were treated with LDLT, and five patients (0.64%) had a history of spontaneous rupture of HCC (Table 1). All five patients were men with a median age of 53 years (range, 49–55). Preoperative imaging showed that these patients had multiple HCC tumors, and the median tumor size was 7.4 cm (range, 3.1–15.3) (Figure 1). Liver function before LT varied as tumors in two patients (40%) were classified as Child-Pugh Class A, one (20%) was Class B, and two (40%) were Class C. One patient (20%) had PVTT on preoperative imaging. Three patients (60%) were treated with TACE or TAE immediately after diagnosing rupture of HCC and subsequently underwent LDLT as elective surgery. Specifically, since patients No.1 and No.4 were newly diagnosed with HCC at the time of rupture, TACE was performed, and the patients were discharged without adverse events. The patients requested LDLT, and the operation was performed within 3–20 months after the rupture day. Patient No.2 had HBV-LC with a history of three TACE sessions and one percutaneous ethanol injection therapy and was waiting for the scheduled LDLT operation date when the tumor rupture occurred. Elective LT was rescheduled and performed 3 days after the rupture, which was treated with TACE. In contrast, two patients (40%) were conservatively treated as active bleeding was indefinite, and the vital signs were stable when the rupture was identified. Patient No.3 underwent hepatic resection (left lateral segmentectomy and S5 tumorectomy of the liver) 1 week after HCC rupture. LDLT was performed ~6 years after the rupture date, while the patient underwent TACE and radiofrequency ablation (RFA) once each. Patient No.5 was diagnosed with HCC rupture when he was admitted for transplantation workup. LDLT was performed immediately after the workup, which was 2 days after identifying the tumor rupture.

**Table 1:** Preoperative data of patients.

**No**	**Sex**	**Age**	**Etiology**	**Child-Pugh class (score)**	**MELD Score**	**Status of tumor (image)**			**Interval from rupture to LT (days)**	**Treatment between rupture to LT**	**MoRAL score**
						**Number**	**Max. Size (cm)**	**PVTT**			
1	M	49	HBV	C(11)	27	Multiple	3.1	(–)	92	TACE	NA
2	M	53	HBV	C(13)	24	Multiple	3.2	(–)	3	TACE	470.8
3	M	58	HBV	A(6)	11	2	8	(–)	2,212	Resection RFA TACE	50.0
4	M	53	HBV	A(6)	10	Multiple	7.4	(–)	609	TACE RFA	78.4
5	M	55	HBV	B(8)	12	Multiple	15.3	(+)	2	(-)	3019.5

*MELD, model for end-stage liver disease; PVTT, portal vein tumor thrombus; MoRAL (model to predict tumor recurrence after LDLT); M, male; HBV, hepatitis B virus; TACE, transarterial chemoembolization; RFA, radiofrequency ablation; LT, liver transplantation; NA, not available*.

**Figure 2:**
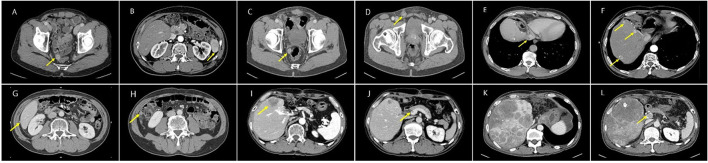
Images of patients No. 2 **(A–F)** and No. 5 **(G–L)** with HCC recurrence. **(A,B)** Rectal serosal, subsplenic lesion, **(C,D)** rectal serosal, right inguinal lesion, **(E,F)** IVC tumor thrombus, intrahepatic multiple HCC, **(G,H)** perihepatic, subhepatic lesion, **(I,J)** intrahepatic HCC recur, portal vein tumor thrombus, and **(K,L)** aggravation of intrahepatic lesions and portal vein tumor thrombus.

The median follow-up duration among all patients was 85.3 months (range, 12.4–182.7). Two patients (40%) experienced recurrence during follow-up (Table 2), and each recurrence-free survival was 4.3–17.0 months after the transplantation. Patient No.5 was diagnosed with a hypermetabolic lesion in the perihepatic area 4.3 months after the operation. The patient was treated with surgical resection and confirmed as peritoneal dissemination of HCC rupture. Another subhepatic metastatic lesion was found 7 months after LT, which was also surgically resected. The patient developed multiple intrahepatic HCC tumors and PVTT that were treated with TACE, but the patient succumbed at 12.4 months after LT (Figure 2). Furthermore, patient No.2 was found with a rectal serosal mass on a regular follow-up CT scan at 17 months after transplantation. Surgical resection was performed and confirmed as HCC metastasis due to peritoneal dissemination. The patient was diagnosed with a similar metastatic lesion at 25 and 43 months after the operation and also underwent surgical resection. Despite treatment with sorafenib and four cycles of TACE for repeated intrahepatic recurrence, the patient succumbed to cancer progression 79.1 months after LT (Figure 2). Therefore, peritoneal dissemination of HCC was detected in two (40%) of five patients who underwent LDLT with a history of spontaneous HCC rupture. Patients with peritoneal recurrence eventually succumbed during the follow-up period.

**Table 2:** Postoperative findings and follow-up after LDLT.

**No**	**Status of tumor (pathology)**					**Op time (min)**	**Estimated blood loss**	**Follow-up duration (month)**	**Recurrence**	**Recurrence-free survival (month)**	**Alive/Dead**
	**Number**	**Max. size (cm)**	**Necrosis**	**Microvascular invasion**	**Edmonson-Steiner grade (worst/most)**						
1	3	4.2	0%	–	III/III	465	N/A	182.7	–	182.7	Alive
2	4	5.7	40–95%	–	III/II	425	3,500	79.1	+	17.0	Dead
3	2	9	0/30%	–	III/III	365	600	85.3 (f/u loss)	–	85.3	Alive
4	>5	5.5	0–99%	–	II/II	400	1,150	87.4	–	87.4	Alive
5	>5	11.5	5%	+	III/II	355	3,650	12.4	+	4.3	Dead

*Op, operation; f/u, follow-up; LDLT, living donor liver transplantation*.

**Figure 3:**
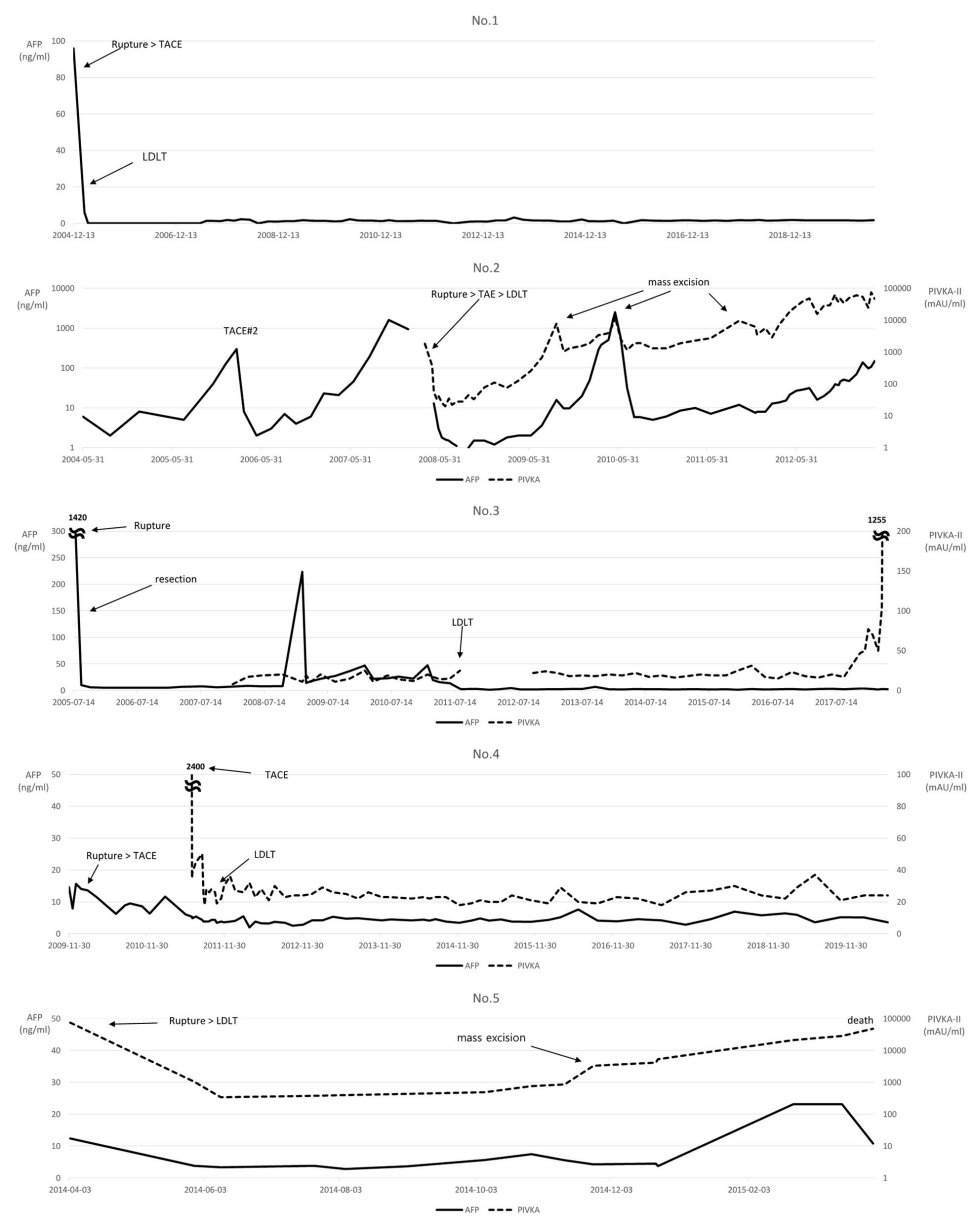
Changes in the levels of serum alpha-fetoprotein (AFP) and protein induced by vitamin K absence or antagonist-II (PIVKA-II).

Serum AFP and PIVKA-II levels were checked throughout the follow-up period (Figure 3). Most patients' AFP or PIVKA-II values were elevated at the time of tumor rupture or tumor aggravation. PIVKA-II values showed a tendency to gradually rise as the disease worsened after recurrence in Patients No.2 and No.5. The MoRAL (model to predict tumor recurrence after LDLT) score (16) before LDLT was calculated in available patients. Two patients showed MoRAL scores that were greater than the cutoff value of 314.8.

**Figure 4:**
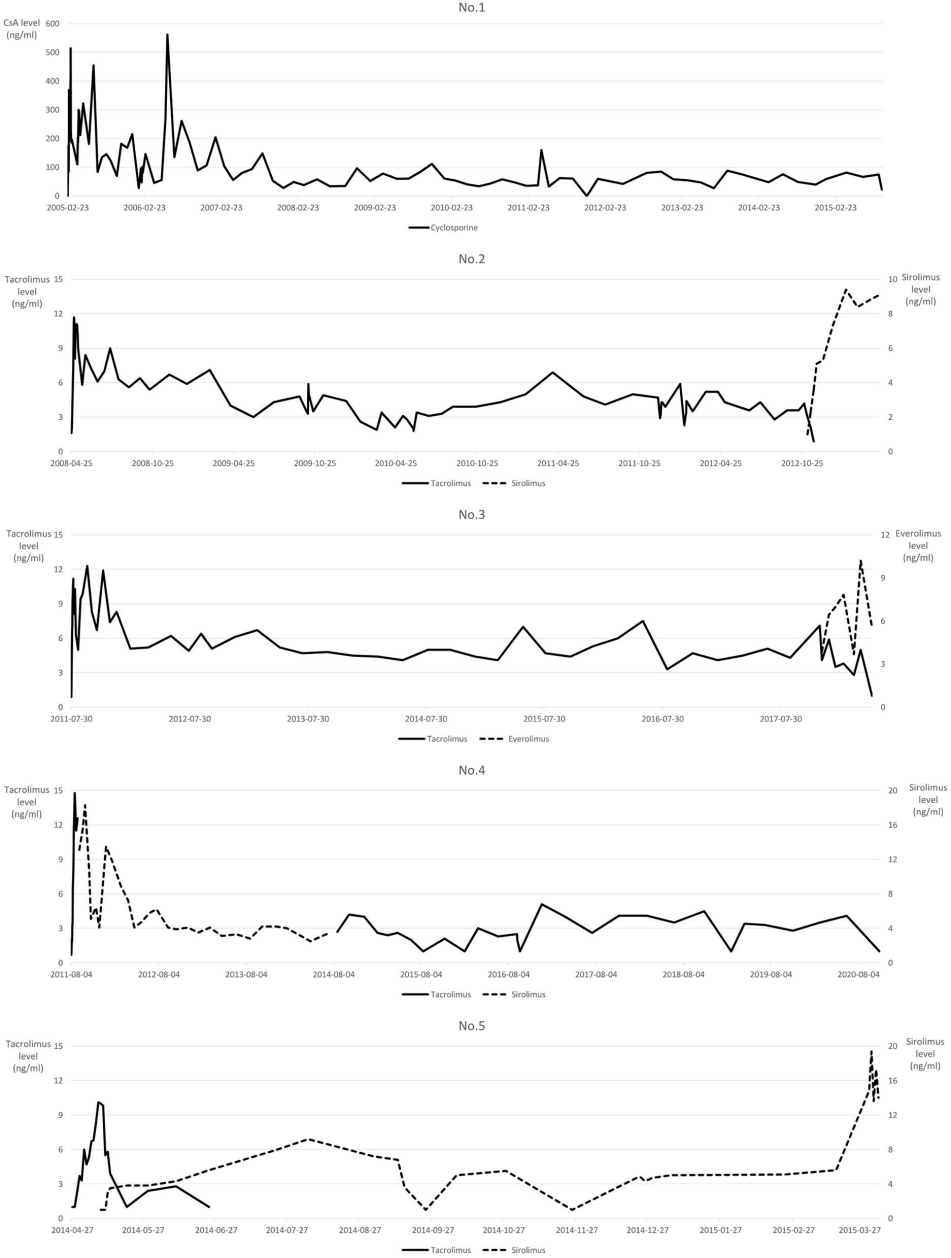
Serum immunosuppressant levels. Non-measurable Tacrolimus values <2 ng/ml are labeled as 1 ng/ml.

Immunosuppression was slightly different in each patient, with similar protocols. Only patient No.1 received cyclosporine A (CsA) as the calcineurin inhibitor, while other patients received tacrolimus, which was switched to mTOR inhibitor at different time points. Tacrolimus was switched to sirolimus in patient No.2 when intrahepatic recurrence was revealed, while everolimus was administered in patient No.3 when he was diagnosed with advanced gastric cancer with peritoneal seeding. Tacrolimus was switched to sirolimus quite early in patients No.4 and No.5 at 12 and 17 days after LT, respectively. Figure 4 shows the serum immunosuppressant levels throughout the follow-up period after LT.

## Discussion

The management of ruptured HCC and its outcomes have been controversial in many studies. According to a multicenter analysis reported by Lee et al. (17), patients treated with staged hepatectomy after HCC rupture showed significantly greater overall survival than those who underwent TACE only. The 1-, 3-, and 5-year overall survival rates of TACE-only group were 40.1, 15.7, and 10.5%, respectively, after HCC rupture. In contrast, the staged hepatectomy group had 1-, 3-, and 5-year overall survival rates of 88.7, 58.4, and 43.8%, respectively. Moreover, the 1- and 5- year overall survival rates of patients undergoing LDLT under the diagnosis of HCC (including both ruptured and non-ruptured HCC) in our institution were 92.8 and 81.5%, respectively (12). In the present study, five patients underwent LDLT for ruptured HCC, of which four survived for >5 years. It was difficult to elucidate statistical significance with the retrospective results of a few patients. However, the results were not insignificant compared to that of staged hepatectomy, palliative locoregional therapies, or LDLT in our institution. Considering that the 5-year survival rate with hepatic resection in patients with non-ruptured HCC is 70–80% and the disease-free survival rate is 30–50% (18, 19), the performance of LDLT in cases of ruptured HCC can be considered relatively good. Although it was difficult to compare directly because the sample size was small and there were many biases to consider, it is worth considering. Nevertheless, further studies comparing the outcomes of LDLT and other treatment methods with larger sample sizes are necessary to clarify this issue.

Chen et al. (15) were the first to report LT in a patient with a history of spontaneous rupture of HCC. Invasion of the diaphragm and right lower lung was identified during the operation for DDLT, which was resected together with total hepatectomy. The patient received postoperative chemotherapy using doxorubicin, along with immunosuppression using cyclosporine, azathioprine, and prednisolone, and he showed no disease recurrence at 23 months after LT. Jeng et al. (13) reported LDLT in a patient with a history of HCC rupture, a case that was beyond the Milan criteria. The patient received LDLT 11 months after the bridging period, while downstaging of HCC with three cycles of TACE and no recurrence was observed 20 months after the operation. Data and outcomes of previous studies on LT after HCC rupture and our present study results are described in Table 3 (13–15, 20). The present study contributes significantly to the field compared to previous case reports because this study had multiple cases treated with a similar protocol at a single center and was limited to patients who received a liver from a living donor. We also provide data on tumor biology, such as AFP and PIVKA-II values throughout the treatment period and other specific data on tumor pathology. Moreover, the follow-up duration was longer than that in existing cases, providing insight into long-term outcomes, such as the 5-year overall survival rate.

**Table 3:** Data of previous reports of LT after HCC rupture.

**Authors**	**Publication**	**Number of cases**	**Etiology**	**AFP (ng/ml)**	**PIVKA-II (nAU/ml)**	**C-P class (score)**	**MELD score**	**Op**.	**EBL (ml)**	**Down-staging**	**PVTT**	**Bridging-period**	**Pathology data (necrosis)**	**F/U** **Period**	**Recurrence**	**RFS**	**O/S**
Chen et al. (15)	2000	1	HCV	241	N/A	N/A	N/A	DDLT	N/A	TACE	–	N/A	N/A	23 mo	-	23 mo	23 mo
Prieto-Puga et al. (14)	2015	1	Alcoholic	N/A	N/A	B(8)	N/A	DDLT	N/A	–	N/A	19 mo	N/A	>9 yr	-	>9 yr	>9 yr
Sadykov et al. (20)	2015	1	HBV	7.5	423	C(10)	10	LDLT	N/A	–	–	3 mo	N/A	70 mo	+	32 mo	70 mo
Jeng et al. (13)	2019	1	Alcoholic	N/A	N/A	N/A	8	LDLT	N/A	TACE	–	10 mo	3.5 cm (100%)	20 mo	-	20 mo	20 mo
Lee et al. (this study)		5	All 5 HBV	+	+	A(6) to C(13)	10–27	LDLT	4 of 5 (600–3650)	4 of 5	1 of 5	0–72mo	+ (size, number, E-S grade, necrosis)	1–5yr (median 85.3 mo)	2 of 5	4–182.7 mo (median 85.3 mo)	12.4–182.7 mo (median 85.3 mo)

*AFP, alpha-fetoprotein; PIVKA-II, protein induced by vitamin K abscence or antagonist-II; C-P, Child-Pugh; MELD, model for end-stage liver disease; Op, operation; EBL, estimated blood loss; PVTT, portal vein tumor thrombus; F/U, follow-up; RFS, recurrence free survival; O/S, overall survival; HCV, hepatitis C virus; N/A, not available; HBV, hepatitis B virus; DDLT, deceased donor liver transplantation; TACE, transarterial chemoembolization; LDLT, living donor liver transplantation*.

This study observed a correlation between surgical outcomes and whether the transplant surgery was performed immediately after HCC rupture. The overall survival and disease-free survival rates were much more favorable when LDLT was performed with a bridging period after tumor rupture. A benefit of treatment with a bridging period is that it can check for peritoneal recurrence. Some studies claim that HCC rupture does not increase the risk of peritoneal seeding (10, 11). However, the issue remains unresolved, as some studies report a greater peritoneal seeding rate in ruptured HCCs than in non-ruptured HCCs (21). Therefore, LDLT should be considered after monitoring for peritoneal recurrence. As mentioned in the report by Jeng et al. (13), another benefit of the bridging period is that it can be used for the downstaging of HCC. Patient No.4 underwent a similar treatment method: multiple rounds of TACE and RFA during the 20 months of bridging period that successfully downstaged the tumor, with concurrent monitoring for extrahepatic metastasis. In patient No.3, HCC rupture was treated with hepatic resection, not TAE, and hepatic resection also showed an equivalent effect as a treatment modality in downstaging the tumor during the bridging period.

There are numerous studies on LT after the downstaging of patients with HCC beyond the Milan Criteria (22). It is suggested that tumor biology data should be included before selecting the LDLT candidates. Optimal protocols for performing post-downstaging LT are unavailable (23), but patients with a history of HCC rupture may also be included in downstaging if other conditions are met.

The MoRAL score calculated using AFP and PIVKA-II levels determines the adequacy of transplant surgery by verifying the probability of recurrence after LT in HCC patients beyond the Milan Criteria (24). Based on the MoRAL score calculated in this study, HCC recurred in patients No.2 and No.5, who had high MoRAL scores. Their scores, 470.8 and 3019.5, respectively, were greater than the cutoff value of 314.8. Other patients with a score below the cutoff value survived without tumor recurrence, suggesting that it may help assess recurrence through biological markers using MoRAL scores before LDLT, even in patients with a history of HCC rupture.

Recent studies have shown mTOR inhibitors to have concurrent immunosuppressive and anticancer effects, and thus they protect the transplanted liver and prevent HCC recurrence (25). However, the benefit of administering mTOR inhibitors in patients with HCC undergoing LT remains controversial (26, 27). In the present study, an mTOR inhibitor was administered at different points during the treatment course in each patient for immunosuppression and antitumor effects. Except for patient No.1, who had an operation in 2005 before mTOR inhibitor benefits were known, mTOR inhibitors were administered in almost all patients in this study. As the mTOR inhibitor was administered after intrahepatic recurrence or detection of *de novo* gastric cancer in patient No.2 and No.3, it was difficult to infer the effect of mTOR inhibitors on tumor recurrence in these cases. Sirolimus was administered relatively immediately after transplantation in patients No.4 and No.5. According to our previous report (28), sirolimus does not decrease HCC recurrence but prolongs overall survival in patients with LDLT beyond the Milan criteria. Therefore, sirolimus may have affected the survival after repeated tumor recurrence in patient No.5. Further research is needed to evaluate the positive effects of early sirolimus administration in patients with advanced HCC.

Other than some of the factors mentioned, which may have affected the outcomes of LT after HCC rupture, we also noted factors that did not affect the surgical outcomes. Since most patients underwent preoperative procedures such as TACE and had multiple HCCs before surgery, it was difficult to assess the effects due to the degree of tumor necrosis on patient survival. In general, spontaneous necrosis of the tumor is thought to be associated with a poor prognostic factor of survival, as it implies aggressive tumor behavior with rapid growth. However, patient No. 5, who did not undergo any preoperative procedures before LDLT, had a low degree of necrosis and poor survival outcome. This may imply that the degree of necrosis may not critically affect patient survival.

Patient No.5 was identified with a portal vein tumor thrombus before surgery, with high serum PIVKA-II levels. Therefore, we hypothesized that the tumor biology would be aggressive and the patient would be at an increased risk of recurrence. However, LT was performed with the patient and donors' consent and request after a sufficient explanation of the risk of recurrence. Macrovascular invasion may not be an absolute contraindication for LT (29), but the patient died 12.4 months after the operation because of tumor recurrence and aggravation. Most patients with PVTT have a poor prognosis with overall survival of 2–4 months with supportive care (30); however, >12 months of survival may not be a poor result in patients in the palliative settings. This suggests the efficacy of LT at the palliative level, which may be a promising method for extending life expectancy even in patients with PVTT or a history of HCC rupture if the risk and prognosis are completely agreed upon. However, even though living donor organs can be viewed as assets of families, further discussion is needed on this decision because transplantation surgery can deeply alter the quality of life of both donors and recipients in terms of both physiological and psychological aspects.

This study has pros and cons compared to previous studies. As shown in Table 3, this study has the largest sample of liver transplants performed in this particular group of patients. Moreover, compared to previous case reports, we presented various pre- and post-operative data, especially focusing on tumor biology. Some limitations of this study include the limited number of patient samples, and the analyses lacked statistical significance, which makes it mostly descriptive. Even though the patients were treated using the same protocol in the same institution, the surgeries' years were different, making the preoperative treatment quite different. Therefore, each clinical course should be checked in detail to suggest future research directions, rather than generalizing the outcomes of survival and recurrence rates. However, owing to the nature of ruptured HCC, it has been difficult to conduct research on a large number of patients. Moreover, since this study does not thoroughly compare LDLT with other treatment methods such as locoregional treatment or other palliative approaches, far more larger studies are needed to obtain solid conclusion about this issue. Thus, a large-scale multicenter study is needed to overcome these limitations.

This study reports favorable outcomes of LDLT in patients with a history of HCC rupture. LDLT could be performed in patients with HCC tumors beyond the Milan criteria after downstaging using various treatment modalities when sufficient information about tumor biology was available. Therefore, patients with ruptured HCC may also be considered candidates for LDLT when establishing LT and postoperative care protocols in advanced HCC.

## Data Availability Statement

The original contributions presented in the study are included in the article/supplementary material, further inquiries can be directed to the corresponding author.

## Ethics Statement

The studies involving human participants were reviewed and approved by the Institutional Review Board of Seoul National University Hospital approved this study (IRB no. 2003-114-1110). Written informed consent for participation was not required for this study in accordance with the national legislation and the institutional requirements.

## Author Contributions

HYL and SKH made the concept and design. HYL, SKH, SyH, and SS acquired, analyzed, and interpreted the data. HYL drafted the manuscript. HYL, SKH, SyH, SS, ESH, J-ML, YC, N-JY, K-WL, and K-SS critically revised the manuscript for important intellectual content. All authors contributed to the article and approved the submitted version.

## Conflict of Interest

The authors declare that the research was conducted in the absence of any commercial or financial relationships that could be construed as a potential conflict of interest.

## Publisher's Note

All claims expressed in this article are solely those of the authors and do not necessarily represent those of their affiliated organizations, or those of the publisher, the editors and the reviewers. Any product that may be evaluated in this article, or claim that may be made by its manufacturer, is not guaranteed or endorsed by the publisher.
